# Patients’ Experiences With the Fit of Virtual Atrial Fibrillation Care During the Pandemic: Qualitative Descriptive Study

**DOI:** 10.2196/41548

**Published:** 2023-01-30

**Authors:** Kathy L Rush, Lindsay Burton, Peter Loewen, Ryan Wilson, Sarah Singh, Lana Moroz, Jason G Andrade

**Affiliations:** 1 Faculty of Health and Social Development School of Nursing University of British Columbia Okanagan, Kelowna, BC Canada; 2 Faculty of Pharmaceutical Sciences University of British Columbia Vancouver, BC Canada; 3 Centre for Cardiovascular Innovation Vancouver, BC Canada; 4 Atrial Fibrillation Clinic Royal Columbian Hospital New Westminster, BC Canada; 5 Department of Medicine University of British Columbia Vancouver, BC Canada; 6 Montreal Heart Institute Université de Montréal Montréal, QC Canada

**Keywords:** atrial fibrillation, virtual care, patient experience, qualitative, communication, quality of care

## Abstract

**Background:**

In-person health care has been the standard model of care delivery for patients with atrial fibrillation (AF). Despite the growing use of remote technology, virtual health care has received limited formal study in populations with AF. Understanding the virtual care experiences of patients in specialized AF clinics is essential to inform future planning of AF clinic care.

**Objective:**

This qualitative descriptive study aimed to understand patients’ virtual AF clinic care experiences during the COVID-19 pandemic.

**Methods:**

Participants were recruited from a pool of patients who were receiving care from an AF clinic and who were enrolled in a larger survey study. A total of 8 virtual focus groups (n=30) were conducted in 2 waves between March 2021 and May 2021. Facilitators used a semistructured discussion guide to ask participants questions about their experiences of virtual care and the perceived quality of virtual care and technology support. Three team members initially open coded group data to create a preliminary coding framework. As the analysis progressed, with subsequent focus groups, the code clusters were refined.

**Results:**

The participants were primarily male (21/30, 70%), aged ≥65 years (20/30, 67%), and college graduates (22/30, 73%). Patients found virtual care to be highly beneficial. Central to their experiences of virtual care was its fit or lack of fit with their health needs, which was integrally connected to communication effectiveness and their preferred virtual care future. Practical benefits included flexibility, convenience, and time and cost savings of virtual care. Virtual care fit occurred for small, quick, and mundane issues (eg, medication refills) but was suboptimal for new and more complex issues that patients thought warranted an in-person visit. Fit often reflected the effectiveness of communication between patient and provider and that of in-clinic follow-up. There was near-complete agreement among participants on the acceptability of virtual communication with their providers in addressing their needs, but this depended on adequate reciprocal communication. Without the benefit of in-person physical assessments, patients were uncertain and lacked confidence in communicating the needed, correct, and comprehensive information. Finally, participants described concerns related to ongoing virtual care with recommendations for their preferred future using a hybrid model of care and integrating patient-reported data (ie, blood pressure measurements) in virtual care delivery.

**Conclusions:**

Virtual care from a specialty AF clinic provides practical benefits for patients, but they must be weighed against the need for virtual care’s fit with patients’ needs and problems. The stability and complexity of patients’ health needs, their management, and their perceptions of communication effectiveness with providers and clinics must be considered in decisions about appointment modality. Patients’ recommendations for future virtual care through use of hybrid models together with systems for data sharing have the potential to optimize fit.

## Introduction

### Background

In-person health care has been the standard model of specialty care for patients with atrial fibrillation (AF). However, there have been steady advancements in technology for remote arrhythmia detection, such as electrocardiogram patch monitoring via mail and other app-based patient heart rate and rhythm monitoring systems, which have been highly effective [1]. However, virtual care has received limited research attention [2,3]. Virtual care has been defined as any interaction between patients or members of their circle of care occurring remotely, using any form of communication or information technology with the aim of facilitating or maximizing the quality and effectiveness of patient care [4,5].

Virtual care was used to a limited extent in AF care [6] before COVID-19, with a few studies showing similar levels of satisfaction between virtual and in-person consultations [2,7-9]. Although the acute phase of the COVID-19 pandemic radically transformed care delivery models to virtual care as the new normal, there continues to be limited research exploring the use of virtual AF care delivery and none from a patient perspective. For example, the European TeleCheck-AF project combining the use of remote app-based heart rate and rhythm monitoring before teleconsultations reported that patients found the app easy to use and install and that it provided a feeling of safety [10], but patients’ experiences with the teleconsultations were not addressed. A Canadian survey study, which was not specific to AF, found that 88% (n=45) of patients who had had a virtual visit with a cardiology health care provider during COVID-19 were satisfied (13% somewhat satisfied, 30% satisfied, and 45% very satisfied) with the virtual format, but there was no in-depth exploration of patients’ experiences or their perceptions of the quality of their virtual care [11].

### Objective

In Canada, structured, integrated, multidisciplinary, and patient-focused care that can be delivered by specialized AF clinics is recommended by consensus guidelines [12], and AF clinics are increasing in prevalence [13,14]. As virtual care is projected to continue following the acute pandemic, the future and sustainability of virtual AF care remain unknown. It is essential to understand the virtual care experiences of patients in specialized AF clinics and their views of deficits and successes with virtual care to help inform future planning of virtual AF clinic care. Therefore, this qualitative study, part of a larger cross-sectional study exploring the AF clinic’s virtual care delivery, aimed to understand patients’ perceptions and experiences of virtual AF clinic care during the pandemic.

## Methods

### Design

Qualitative description was used to produce a detailed and nuanced interpretation that stayed close to the participants’ data and their everyday language [15]. Consistent with the constructivist paradigm, which views reality as socially constructed, it allowed for an in-depth understanding of patients’ experiences with specialty virtual care. The conduct and reporting of the study followed the Consolidated Criteria for Reporting Qualitative Studies guidelines for qualitative research reporting [16].

### Ethics Approval

This study received joint approval from the University Behavioural Research Ethics Board and the Health Authority (certificate #H19-03601).

### Setting

This study was conducted in partnership with the largest tertiary urban-based specialty AF clinic in Western Canada. The clinic is 1 of the 5 provincial AF clinics and serves approximately 1900 patients on average every year. The clinic is provincial in scope and comprises a multidisciplinary team of nurse practitioners, pharmacists, registered nurses, cardiologists, and electrophysiologists. The mandate of the clinic is to provide specialized care for patients with newly diagnosed or established AF or atrial flutter, focusing on acute or short-term interventions, chronic disease management, and advanced procedural or electrophysiological care such as ablation. Once a patient’s treatment is optimized (usually within 6-12 months), patients are returned to their primary care clinician for ongoing follow-up. In March 2020, the clinic implemented COVID-19 protocols that restricted appointments to virtual mode only, and as of January 2023, the AF clinic continues to restrict all appointments to telephone, except when providers request in-person appointments.

### Sampling and Recruitment

The study participants were recruited from a pool of patients aged >18 years who had consented to a larger study. For the larger study, the booking clerk initially notified all eligible patients about the study using scripted communication (by email or mail) and that a member of the research team would be contacting them. Patient contact information was shared with the research team through secure file transfers. Subsequently, a research assistant (a physician or a licensed practical nurse) who had no previous relationship with the participants contacted the patients by telephone.

**Figure 1:**
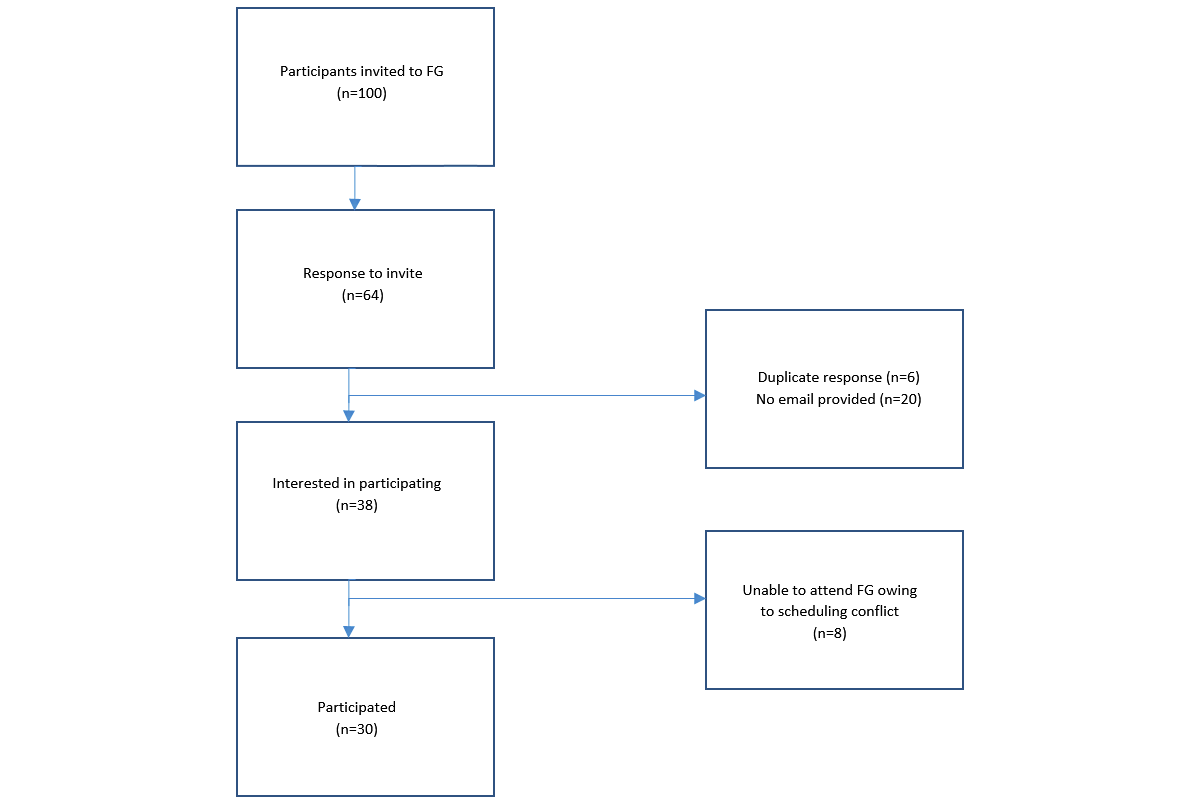
Recruitment flowchart. FG: focus group.

Focus group recruitment occurred during recruitment for the larger study, with researchers sending patients who had consented and completed the survey (as of March 2021, n=100) an email invitation to participate in the focus groups. The email included a link to indicate availability from 4 midweek dates and times (Figure 1), and if in conflict, their interest in possible future focus group participation. Interested patients received an email including a link to a web-based consent form and focus group questions in preparation—a strategy recommended to avoid “GroupThink,” or thinking like other group members, that may not reflect individual thinking. When no additional insights were provided by the last focus group, no further focus groups were conducted [17] as agreed by the investigators.

### Data Collection

Consistent with the qualitative description, focus groups were an efficient way to obtain a broad range of information (who, what, and where) about the nature and shape of virtual care experiences [18] through facilitated discussion. Focus groups allowed participants to exchange ideas and information; react, build on, stimulate, or challenge each other’s thinking to generate a range of perceptions, insights, and experiences about virtual AF care; and avoid acquiescence and transference effects while maintaining rapport [19]. Consistent with our purpose, we used semistructured questions and facilitated the group interaction to productively generate content while paying careful attention to the general amount of agreement and disagreement between participants as well as the level of emotion (eg, enthusiasm, indifference, and dispassion) [19].

A total of 8 focus groups (ranging in size from 2-5 participants) were conducted over 3 months (March 2021-May 2021), with participants joining from their homes or workplaces using Zoom videoconference software (Zoom Video Communications). Trained facilitators (KLR, RW, and LB) followed a semistructured interview guide (Multimedia Appendix 1), asking questions about participants’ experiences of virtual care and their perceptions of virtual care quality and technology support. Facilitators used prompts and probes to encourage greater clarification, elaboration, and depth in detailing their experiences. Facilitators also asked contextual questions such as participants’ distance from the clinic and employment status. Only researchers and participants were present during and after the focus groups to take field notes of the focus groups. The recorded focus groups lasted 1 to 1.5 hours. The team debriefed following each focus group to discuss any new insights gained and from whom and determined the need for a second wave of recruitment 1 month following the first wave to maximize sample diversity and to explore new and emerging ideas. It was anticipated that additional focus groups would expand the opportunities to recruit women and visible minority individuals who were underrepresented in earlier groups. In addition, data sharing emerged in early focus groups, and it became a more focused line of exploration in subsequent focus groups.

Additional information including demographics, health history, and consultation type (follow-up, new, or ablation) were extracted from the participants’ previous survey responses. Geographic distance from the clinic was determined using a combination of previous survey responses and focus group discussions.

### Data Analysis

NVivo-transcribed (QSR International) focus group audio recordings were checked for accuracy against recordings (SS) and subsequently analyzed thematically. The transcribed data were coded following each focus group. Data from the first 2 focus groups were initially open coded by 3 team members (KLR, LB, and SS) who met to discuss and cluster similar codes into themes and subthemes to generate a preliminary coding framework. A research assistant used the preliminary framework to code the data using NVivo (version 12; QSR International). As the analysis progressed with subsequent interviews, codes were created, modified, and in some cases, collapsed and renamed. NVivo was used to recode the data. Maintaining an audit trail and involving multiple team members in the data analysis were used to enhance trustworthiness of the data. The researchers engaged in ongoing reflexivity and reflection to avoid influencing the research process and data analyses. Demographics and health history were analyzed descriptively using R software (R Foundation for Statistical Computing).

## Results

### Overview

In total, 30 patients participated in the focus groups. Participants were primarily male (21/30, 70%), ≥65 years (20/30, 67%), and living within the clinic’s greater metropolitan area (21/30, 70%; Table 1), similar to the clinic demographics in which patients were primarily male (4004/6566, 60.98%), ≥65 years (3742/6566, 56.99%), and living within the clinic’s greater metropolitan area (4793/6566, 72.99%). Male participants in the sample were significantly younger and had higher incomes than female participants (Table 1). Patients had, on average, 2 chronic diseases inclusive of AF and primarily rated their health (19/30, 63%) and mental health (27/30, 90%) as good or excellent. The reasons for virtual appointments were follow-up (16/30, 53%), new consultation (8/30, 27%), and ablation (6/30, 20%), and they were primarily conducted by telephone (27/30, 90%).

Patients often described their experiences and perceptions of virtual care relative to in-person care. They lauded the practical benefits of virtual care. Despite perceived benefits, central to patients’ experiences of virtual AF care was its fit or lack of fit with their health needs, which was integrally connected to communication effectiveness and their preferred virtual care future.

**Table 1:** Participant demographics.

		Overall (N=30)	Female (n=9)	Male (n=21)	*P* value^a^	
Age (years), mean (SD)		66 (9)	73 (6)	63 (8)	.004	
**Age group (years), n (%)**						.01
	<65	10 (33)	0 (0)	10 (48)		
	≥65	20 (67)	9 (100)	11 (52)		
**Marital status, n (%)**						.15
	Common Law	1 (3)	1 (11)	0 (0)		
	Widowed	1 (3)	1 (11)	0 (0)		
	Single (never married)	4 (13)	1 (11)	3 (14)		
	Married or remarried	24 (80)	6 (67)	18 (86)		
**Ethnicity, n (%)**						>.99
	Asian	3 (10)	1 (11)	2 (9.5)		
	White	27 (90)	8 (89)	19 (90)		
**Education, n (%)**						.66
	Completed high school	2 (7)	0 (0)	2 (10)		
	Some college	3 (10)	0 (0)	3 (14)		
	College or university graduate	22 (73)	8 (89)	14 (67)		
	Other (specify)	3 (10)	1 (11)	2 (10)		
**Income (CAD $; CAD $1 = US $0.75), n (%)**						.002
	<25,000	3 (10)	2 (22)	1 (5)		
	25,000-50,000	4 (13)	4 (44)	0 (0)		
	51,000-75, 000	6 (20)	1 (11)	5 (24)		
	>75,000	17 (57)	2 (22)	15 (71)		
**Housing, n (%)**						.054
	Apartment or condo	7 (23)	0 (0)	7 (33)		
	Detached home	22 (73)	8 (89)	14 (67)		
	Other (specify)	1 (3)	1 (11)	0 (0)		
**Living arrangement, n (%)**						.17
	Live alone	4 (13)	1 (11)	3 (14)		
	Live with children	5 (17)	0 (0)	5 (24)		
	Live with other family members	3 (10)	0 (0)	3 (14)		
	Live with partner	18 (60)	8 (89)	10 (48)		

^a^Wilcoxon rank-sum test; Fisher exact test.

### Practical Benefits of Virtual Care

Participants were unanimous in their opinion to use virtual care during COVID-19 to ensure the protection of both patients and staff. Patients described several practical benefits of virtual AF care, including convenience, cost and time savings, reduced stress, and opportunities for family participation. The participants described virtual care as less disruptive and more convenient than in-person care. For example, rather than the interruption of going for an office visit, they could easily integrate a virtual visit into their daily lives, whether it was during a workday, a family vacation, or while running errands. A participant described the freedom and flexibility of phone use when scheduling appointments ahead of time:

...you have the freedom to keep doing what you’re doing and then just set that time aside when you’re expecting a phone call.

P6, male aged 68 years, follow-up

Participants expressed that unlike in-person visits, virtual appointments did not keep the patients waiting in a crowded waiting room reading magazines and interacting with others (and being exposed) if the provider was late, which in some cases could be quite substantial (eg, an hour).

All the patients lauded the benefit of virtual care in terms of time and cost savings. For participants who lived in rural areas and at significant distances from the AF clinic (approximately 150-2400 km), virtual care reduced travel, ferry, parking, and in some cases, accommodation costs. A participant who lived some distance from the clinic (approximately 200 km) and had a lower income elaborated as follows:

I live over in [a rural community], and over the 10 years I’ve had atrial fibrillation, I’ve had made anywhere from two to four checkup visits to [the clinic] every year to see [cardiologist], who is an absolutely charming man. So I didn’t mind going for the checkups. I could usually squeeze in a visit to friends and other things. However, a lot of the times the checkups are very routine and mostly done by a student. So I could have just as easily done that on Zoom and I would save myself a couple of days of time each visit. So I think Zoom, for me it looks like a technology that I hope stays around for a while.

P13, female aged 81 years, follow-up

Participants living within the metropolitan area with travel times up to 1 hour similarly highlighted the benefit of virtual care:

...it’s just so much more convenient that you don’t have to drive to the office.

P26, male aged 67 years, follow-up

Having family members to be able to attend appointments that otherwise they may not be able to attend in person was also viewed by patients as an added benefit to the virtual consultations.

### Fit of Virtual Care With Patients’ Health Needs and Problems

#### Overview

Patients often gauged the quality of their virtual care experiences according to its fit in meeting their health needs and problems. When they perceived that the virtual modality suitably aligned with their various needs, it was a fit; however, when they were not aligned, they regarded it as a suboptimal fit. They described fit as dependent on the nature and complexity of their health problems, stability of their AF, and extent of decision-making related to their disease management.

#### Fit

When the health problem or need was simple, uncomplicated, straightforward, and easy to resolve, patients deemed virtual care to be a good fit. Fit extended to the management of the need, which included medication prescription refills, laboratory requisitions, quick questions, and other short inquiries, and this was evident in the sentiments of 2 participants:

So if it’s mundane stuff that doesn’t require an in-depth discussion, I would just as soon be on Zoom.

P9, male aged 69 years, new consultation

I think it [virtual] works very well for things like medications and just the questions

P27, female aged 74 years, ablation

Another patient echoed that a virtual appointment works as long as the providers have all the information they need:

EKGs and Holter monitors and all the other information is coming from other places. So you don’t really need to be there.

P6, male aged 68 years, follow-up

#### Suboptimal Fit

In contrast, patients viewed a virtual appointment as a suboptimal fit for a new or serious AF diagnosis, for a changing health situation (eg, concerning symptoms), when making important health decisions, or when experiencing postprocedural complications (eg, after the ablation). They described these complex situations as requiring more in-depth discussions, such as one participant who described serious diagnosis-related discussions as being more appropriate for in-person and not Zoom:

If I’m sitting down with a physician and I’m discussing a diagnosis and some serious issues as to choices that must be made, I think I’d prefer a face-to-face discussion.

P9, male aged 69 years, new consultation

Another patient echoed the need for in-person appointments to facilitate compassionate care when dealing with a new issue or diagnosis:

If you’ve got some like a new issue or a previously undiagnosed like I think that having that in person is probably going to have a bit more comfort, to it. I think you have to think about the...I don’t want to say compassion, you just have to be able to have the other person and the tone and the voice and just the physical body language would make, I think myself feel at ease if, you know, obviously that’s something really serious and you can read it in a doctor’s body language or even in a nurse’s body language. I think that those visual cues you definitely can’t have over the phone or through even a video perspective. Right? So that’s something that would help from a patient, even from a long-term patient, just having that compassion and just being able to hear that from a doctor.

P20, male aged 48 years, ablation

Virtual care was seen as a suboptimal fit for meeting the needs of newly diagnosed patients or patients new to the clinic. Patients described new patients to the clinic as lacking a context for their care and established connections with the clinic team and whose condition was often unstable. For example, patients described the impact of virtual care on new patients’ orientation to the team, facilities, and workflow and trust gain as follows:

...the people that you’re putting your life in their hands.

P27, female aged 74 years, ablation

One of the sequelae of the pandemic for newly diagnosed patients (9/30, 30%) was receiving all their care virtually and never having seen the physical space that housed the clinic slowing their familiarity with the workflow and making the clinic appear as a “...giant black box...” New patients expressed a strong and unmet need to know the treatment pathways and options, as expressed by a male participant in his mid-50s:

If I’m coming into the system, I want to know what the paths are to get me through it. And I never had that sense. I had to create it myself.

P14, male aged 58 years, new consultation

Virtual appointments were not always seen to be a good fit for information exchange and patient education, particularly when patients needed more explanation and education. Although several participants were satisfied with the explanations about pending procedures (eg, ablation) and opportunities for questions during virtual appointments, others found the virtual modality to be more limited in this regard. Unlike in-person appointments, virtual care was found to be restrictive by several patients in situations when providers communicated information that was complex, “over my head,” and “very technical” in nature. They expressed their need for simplified explanations (“putting in more of a layperson’s terms”) and the use of supplementary means, such as visual diagrams to help them understand their health condition, and recounted their specialists’ drawing diagrams that were easier to do in person than over the phone.

### Communication

#### Overview

Communication was a critical facet of patients’ overall perceptions of their virtual care experiences and its fit in meeting their needs. There was near-complete agreement from participants who found virtual communication with their providers acceptable in making them feel “cared for” and having their needs and questions addressed and not constrained by time. For example, one participant described their virtual care as follows:

I’ve had Zoom calls and telephone calls and I have felt surprisingly well cared for without seeing anybody in person. I felt like everything was covered in the appointments and that people had time for me

P22, female aged 67 years, ablation

Patients were variable in their use of specific virtual modalities, with some using the phone exclusively, whereas others used a combination of phone and Zoom; some had a choice of modality, whereas others did not. The patients described two specific areas of communication that had an impact on their overall virtual care experience: (1) provider-patient communication effectiveness and (2) follow-up communication with the clinic. Although virtual communication with the clinic and providers was highly effective overall, patients also relayed the challenges they experienced.

#### Provider-Patient Communication Effectiveness

Patients varied in their perceptions of virtual communication effectiveness with their providers. Patients described communication effectiveness as dependent (1) on their ability to focus and adequately communicate their needs and concerns and (2) on the provider’s ability to listen, interpret the information, and act on it to address their concerns. A 72-year-old female patient who had a telephone appointment captured these vital elements of effective communication:

My observation is that virtual care is really only as good as a patient’s ability to communicate issues and successes they may have had. And it’s also only as good as the provider’s ability to listen and in the end, interpret.

P1, female aged 72 years, follow-up

Some patients who could describe their situation and symptoms, and how they were feeling and who had the provider interpret the information found the virtual modality very effective and a good fit in addressing their concerns. A participant who had fluctuating heart rates was very pleased with the communication by phone appointment in resolving her problem:

Dr. called me up and I discussed with him my situation. And he gave me he said, you know what? I think your medication is a little too much...So he said, you know what we’ll adjust it, he said try it. And then if there’s a problem, get back to us. And voila, it disappeared. Whatever problems I had, I thought, my goodness, this is really good, even though it was a phone call, right? It was a phone call. I described what I was feeling and the situation. And he said he totally understood what I meant because he had all the paperwork in front of him, all my tests previously.

P23, female aged 74 years, follow-up

Other patients found virtual care to be a suboptimal fit in communicating their needs and concerns compared with in-person care. Without the benefit of in-person physical assessments and having their providers look, listen, or feel, they lacked confidence, reassurance, and validation of their symptoms, what they were feeling, and their symptom analysis. A participant expressed self-doubt about her communication adequacy over the telephone:

I did feel that quite a lot initially because I never did have an in-person consult. So I was a little bit nervous initially that I wasn’t being seen and having my blood pressure done. And somebody listen to my heart and all that kind of physical stuff because I was having to describe my symptoms. And, you know, it’s I wasn’t sure I was covering everything and if anything was being missed. So, yeah, I was a little concerned that I wasn’t being actually physically seen some of the time

P8, female aged 65 years, new consultation

Furthermore, without the hands-on examination, patients doubted and questioned whether their descriptions of signs and symptoms were correct, whether they were communicating the necessary information, or whether they were correctly judging the symptoms that were the most relevant (eg, leg swelling) to share with their providers:

The only disadvantage, I think, is the lack of actual hands-on examination just to, I guess, reassure you that what you’re describing [over the phone]. Is this correct?

P8, female aged 65 years, new consultation

Another patient expressed not receiving enough information during the phone appointment and wondered if he did not ask the right questions. In some cases, patients tolerated symptoms such as leg swelling rather than disclosing them during the video visit:

I just have to judge for myself and put up with it

P19, female aged 82 years, new consultation

This self-doubt about the adequacy of their communication left patients concerned that things were being missed.

At the same time, participants were also concerned that virtual care limited their providers’ ability to assess and interpret their issues. They questioned whether their providers’ interpretation of their clinical symptoms would be the same over the telephone compared with an in-person visit:

[During in-person visits, a provider could] take your pulse and throw you on a Holter monitor or monitor you for a period, they might not come to the same conclusion.

P15, male aged 54 years, follow-up

In some cases, patients found the virtual back and forth with phone calls and emails that did not allow providers to “see” for themselves so inadequate and inefficient in resolving the issue, that they gave up and resorted to an in-person appointment. A patient who found that talking about his symptoms on the telephone did not work concluded as follows:

I think that if there are clinical signs, it’s important to actually be seen in person.[P27, female aged 74 years, ablation]

Patients’ activities during telephone appointments influenced their perceptions of the adequacy of their communication with providers. Patients who treated their virtual appointment like an in-person appointment and prepared ahead of time by making notes optimized communication:

...stay[ing] home to dedicate that time to the phone call...I just have to do that. I can’t do these things while I’m being distracted.

P8, female aged 65 years, new consultation

In contrast, patients who took phone appointments while multitasking (working, driving, or grocery shopping), whether by choice or owing to provider delays, were distracted and found it difficult to focus on the appointment. A patient who had telephone appointments during work hours reported suboptimal communication:

You are not concentrating during work because it’s during your work time...I am distracted by millions of things, you know, and you can concentrate when you go to the office [Dr’s], you get this personal touch, like maybe a little bit more attention to detail and you don’t forget things so.

P17, male, aged 50 years, follow-up

Patients who had used a combination of telephone and Zoom described the greater connection and engagement with providers using Zoom than telephone:

I still feel more connected, through the actual zoom call. And I think it allows you to probably get everything over because you kind of fully engage with that person.

P24, male aged 49 years, follow-up

#### Follow-up Communication With the Clinic

Patients had mixed responses regarding the adequacy of follow-up communication with the clinic in meeting their needs in a timely and efficient manner. Some patients experienced few to no problems and found that the clinic was highly responsive to the immediacy of their needs. One patient appreciated the clinic’s rapid response to their AF compared with a 2-month wait for an injured hip ultrasound:

With your heart, you can’t you can’t kind of wait. So you need to be able to talk to somebody really, really fast. And so I appreciated the responsiveness. I think that’s come out of this. I’d trade responsiveness for the in person. In terms of quality of care, I guess.

P4, male aged 57 years, follow-up

Another patient similarly described a timely response to his informational needs as follows:

I’ve never had any trouble getting, talking to somebody, you know, within a day or two or getting some information, but end up talking more to the nurse practitioners and pharmacists than I actually do the doctor. And they know their stuff

P6, male aged 68 years, follow-up

Other patients encountered challenges with follow-up communication, including unmet expectations about clinic-initiated follow-up appointment scheduling and inefficiencies in their self-initiated efforts in reaching clinic staff for various reasons. Some patient participants described anticipating clinic-initiated follow-up communication about treatment options following an initial consultation or scheduling a 6-month appointment after ablation, but such communication had not occurred. A female participant who lived the farthest from the clinic described her uncertainty about who initiates the communication for a follow-up appointment:

And so I guess that’s one thing that I would say is maybe not as clear for those of us who aren’t more on site than others. And that is who initiates the calls. So, as I say, I’ve waited.

P1, female aged 72 years, follow-up

The reciprocal challenge for other participants was their unproductive self-initiated efforts in accessing clinic staff when they wanted to discuss changes in their health situation such as postprocedural complications, failed treatments (cardioversion and ablation), or following an emergency department visit for an AF episode. They did not know whether they should contact the clinic, who to contact, or how. One participant who was trying to follow-up after an unsuccessful cardioversion in the emergency department said of his efforts at calling:

...then you’re kind of in the feedback loop trying to get a hold of somebody.

P15, male aged 54 years, follow-up

Some participants, following ablation or with new symptom onset, found that they had to be more persistent in making their needs known when using the telephone, as one participant voiced the following:

I have to do a little more poking and following up to kind of make the next step happen.

P4, male aged 57 years, follow-up

### Preferred Virtual Care Future

Looking to the future, patients were highly supportive of the continued use of virtual AF care but were concerned that it might become usual care. Their concerns stemmed from perceived challenges with scheduling in-person appointments and with expanding practices and the potential for patients being underserved. One patient whose in-person visit with the nurse practitioner expedited ablation and cardioversion expressed this concern:

But my concern is that. If the more it becomes the norm, the more when you actually do want to see him [physician], it’ll be harder to do. So that’ll be my big concern. So I’d be very disappointed to see virtual appointments become the norm such that you can’t get in to see somebody.

P21, male aged 71 years, ablation

One participant voiced concerns about provider availability:

I can see that that’s my only trepidation with virtual care, is that if doctors get more [patients], a bit more busier, they’re going to have less time for some of their oldest and most long term patients.

P20, male aged 48 years, ablation

Participants addressed their concerns about the future by offering suggestions and recommendations for optimizing fit of virtual care in meeting and managing their overall care needs. Patients had 2 primary recommendations for optimizing fit: use of a hybrid model of care and integration of patient-reported data in virtual care delivery.

### Use of Hybrid Model of Care

As patients projected to the future, they advocated a mix of in-person and virtual appointments and not just virtual care:

Virtual care is great, but there’s always a place, there’s always a place for it and there’s always a place for in-person visits. So it has to be a mix. Definitely

P17, male aged 50 years, follow-up

Patients described specific uses of a hybrid model with the combination of in-person and virtual visits depending on the newness of their AF diagnosis, stability of their condition, and treatment-related issues. They preferred a predominantly virtual approach with periodic in-person visits when they were more comfortable with AF and their AF was stable but preferred in-person visits during the period of initial or early diagnosis and during periods of disease instability.

Patient capacity was an important consideration in participants’ advocacy for a hybrid virtual AF model. They identified several barriers limiting patient capacity that would need to be addressed to prevent exclusion of some patients with AF. The barriers included patient age, technology literacy and comfort, and memory capacity. Participants suggested that patients who were uncomfortable using virtual modalities might opt not to receive care at all and the potentially distracting nature of virtual care was unsuitable for those with memory loss. Furthermore, they viewed patients who lacked infrastructure, such as inadequate cell signal or internet access and privacy and security issues, as a disadvantage in using virtual care.

In describing the hybrid model, patients thought it was important that they have an in-person option with the suggestion that the choice of virtual versus in-person approach needed to rest with the patient, and there needed to be clearly laid out expectations agreed on by physician and patient for the virtual aspect of the hybrid model:

I wonder if it makes sense, like I’m not sure if it’s there at this point, but if there is something like a patient’s rights or something, where there’s something where both doctors and patients kind of agree in this kind of virtual care, what’s to be expected? It’s just mainly setting expectations. I think to make it easier on both sides...so if there is some sort of way of getting expectations set or some sort of, you know, patient, I don’t want to say patient rights because that seems very legal and stuff, but it’s just an understanding and that we’re both sides are both going to try and do our best to make sure that the patient’s interests are still being protected and being looked after. That’s all we really want as patients.

P20, male aged 48 years, ablation

### Integration of Patient-Reported Data in Virtual Care Delivery

As patients considered continuing the use of virtual care and optimizing its fit with their needs, they described making better use of the biometric data they collected. Several patient participants described actively tracking, monitoring, and recording a range of biometric data, such as blood pressure, pulse, oxygen levels, wearable 6-lead electrocardiogram, and weight. They tended to collect these data using Apple Watches (Apple Inc) and blood pressure cuffs (self-measured or measured at a pharmacy) and used both paper (eg, Excel spreadsheet) and electronic approaches to record.

Patients described using the data they collected both to share with their providers and to give them a sense of personal control in self-managing their AF, as one patient described as follows:

Well, what happens is I know that I have it on Omron. I have just transferred it to my phone. I have all my records. I can tell her [provider] whether she wants to know, you know, and that is really good. That is valuable for me too. I used to write on these cards at the drugstore. A spare piece of paper.

P19, female aged 82 years, new consultation

Another patient described self-monitoring to detect abnormal readings and potential problems to self-initiate contact with providers as needed:

So having that device at home is very reassuring because I can if I’m having problems, I know immediately that I should contact someone.

P13, female aged 81 years, follow-up

However, patients raised privacy concerns regarding sharing these data with the clinic through insecure email or faxes and requested a secure way of transmitting their health information.

Other patients contemplated the purchase of wearables or other devices that would allow them to transmit real-time biometric data to the AF clinic as a complement to remote care:

One of the things since I had my ablation in the end of December this year, I still have brief episodes of irregularity, my heart rate and it is possible to go to a local lab and have an EKG done because I have a standing order, but however, it’s not that easy to do. And also these events tend to be quite short lived sometimes. And I’m wondering about the use of some of these new technologies that have been developed recently, like the AliveCor devices that connect to your phone and they can actually do a six lead ECG. And I was thinking that I would maybe purchase one of these things so that when I have one of these episodes, I could make a record of it and send it to the clinic. And I’m thinking something like that might be extremely useful for this kind of remote care.

P5, male aged 73 years, ablation

Patients highlighted the importance of sharing the information they were tracking with providers. One participant spoke of the benefit of such sharing:

The more information that we track ourselves and that we make available to the cardiologist is to our benefit.

P18, male aged 63 years, ablation

However, despite their data tracking, patients acknowledged limited use of the data in their care. The patients described a need for integrating their existing monitoring practices with the AF clinic:

It would be good if the AF Clinic had some way of getting some of these metrics in into their system. As we are recording all of the time, I’m always recording. I know exactly what my blood pressure is all the time. I know what my heart rate is. I record my heart rate all of the time. If I have a spike, I know immediately all of that stuff. And it’s because of the wearable technology. And I do that for myself because I want to know myself. But it would be interesting if they had some way of using those data in the clinic as well.

P30, male aged 69 years, follow-up

## Discussion

### Principal Findings

Patients described the benefits of virtual care, including convenience, time and cost savings, reduced stress, and opportunities for family participation. However, central to their experiences with virtual care was its fit with their needs that was integrally connected to communication effectiveness and their preferred virtual care future. Patients considered virtual care a fit for simple, uncomplicated, straightforward, and easy-to-resolve issues but a suboptimal fit for new, changing, and complex issues such as important health decisions and postprocedural complications. Patients gauged fit according to the effectiveness of their communication with providers and the clinic. Without the benefit of in-person physical assessments, patients experienced uncertainty, self-doubt, and lack of confidence in communicating their needs appropriately. Finally, patients’ preferred virtual care future to address their concerns about virtual care becoming usual care was as a hybrid model, with ongoing access to in-person care, while optimizing the integration of electronic data sharing into routine practice.

### Comparison With Previous Work

Findings from this study show that overall, patients were positive about their experiences with virtual AF care. In their review of remote cardiology clinic visits during COVID-19, Mishra and Edwards [20] found evidence for the potential of telemedicine to be used to adequately address cardiac conditions such as AF and cited a study that revealed that internet and technology access were not significant barriers to telehealth use [21]. However, the patients with AF in our study expressed some concerns and reservations about virtual care fit with their needs and the impact on communication with their providers.

Consistent with previous research with both cardiac and noncardiac populations [20], patients described many practical benefits of virtual care, such as flexibility, convenience, and time and cost savings. Patients also expressed feeling cared for during virtual visits by members of their care team. However, patients raised concerns about access to in-person visits if virtual visits became usual care rather than augment them and worries about whether communication using virtual visits can be as effective as in-person visits. A US survey of American households found that of 42% of households where a family member had used telehealth, 64% would have preferred an in-person visit despite high satisfaction (82%) [22]. A unique finding of this study was the issue of distraction during virtual visits, including conducting them while a patient was driving. There are few recommendations to guide virtual cardiac care in Canada [6], and guidelines for both patients and providers are needed regarding timeliness (eg, responsiveness and being on time), safety, and ways to promote effective communication during virtual visits.

Patients supported virtual care’s fit for small, quick, and mundane issues (eg, medication refills) but found it suboptimal for new and serious issues that are more appropriate for an in-person visit. Patients’ perspectives aligned with a survey of Canadian cardiologists (n=26) who identified the need for in-person visits for patients who were very sick, had communication challenges, required physical assessment to inform care options, required hands-on tasks, or were new patients [11]. National and provincial virtual care policy and guidelines direct care providers to limit virtual encounters to those requiring only history, gross inspection, or data that patients can gather with cameras or other devices. According to these guidelines, any new or significant symptoms require in-person care rather than virtual care [23]. Although patients thought they should be able to choose the modality for their appointments, the physician’s clinical judgment must also be considered, and this collaborative decision-making is important to consider in negotiating patient and provider virtual care expectations.

Patients suggested greater sharing of patient-reported data with their providers to enhance the benefits of virtual care visits and to better meet their health needs. Jamieson et al [24] regarded virtual care as creating the preconditions for truly empowered patients and patient-centric care—the equivalent of other life activities such as banking and shopping. Such data sharing has the potential for expanding the scope of virtual care. Patients in this study gravitated toward a hybrid model of AF care and made several recommendations for optimizing care using this model. For example, patients recommended supplementing their care with the use of tools and technology to send their providers biometric data. Similarly, in a survey of Canadian patients who had had a virtual visit with a cardiology health care provider, 72% of patients preferred a hybrid model, with 68% indicating interest in using an electronic tool (eg, email or mobile app) to share nonurgent health information with their health care provider [11]. Patient satisfaction with virtual care was high in a study that had patients upload vital signs (heart rate, blood pressure, blood glucose, weight, and temperature) to the virtual platform before their face-to-face video call with the clinical nurse specialist at the technology-enabled arrhythmia clinic [7].

The patients also raised concerns about the effectiveness of virtual communication with their providers. To date, little is known about the effectiveness of patient-provider communication during telemedicine or virtual encounters [25], generally or specific to cardiovascular care [20]. However, evidence has shown that interventions targeting patient-provider communication improve population health, patient and provider experiences, and costs [26]. Study participants emphasized the importance of effective communication—the patient relaying the information accurately and comprehensively and the provider receiving and interpreting it [27]—but often felt a greater weight of responsibility, lack of confidence, and limited validation in virtual communication with their specialists. Similarly, a study on patients’ contributions during virtual gastrointestinal consultations found that patients assumed increased agency in their contributions, as few were explicitly doctor driven [28]. Frankel and Beckman [29] described patients and providers as relational units with shared responsibility in coproducing more efficient and effective interactions. These interactions can be enhanced by improving patients’ health literacy through digital approaches to education [30] and self-management support [31].

Some of the participants’ communication concerns may reflect the high use of the telephone (27/30, 90%) as the primary modality in this study, and such use is consistent with the finding from another Canadian study of virtual cardiology care [11]. Telephone affords only the use of audio communication without the benefit of visual cues that play an important role in complementing the verbal message, especially when virtual care does not permit physical and hands-on examination, and compared with virtual care, it has been linked to lower-quality patient-provider virtual communication [26]. Furthermore, evidence suggests that videoconference may offer improved initial diagnostic accuracy compared with the telephone [32]. Virtual care practice resources and toolkits developed for Canadian physicians that have emphasized patients’ technological readiness or preparation for a virtual video appointment [23] may need to be balanced with consideration of communication issues relevant to both patients and providers. Patients also experienced some communication challenges with the clinic more generally when they were seeking to initiate contact for a variety of reasons but particularly in booking follow-up to procedures, when they relapsed, or had unexpected health events. A directory made available for patients being seen at the clinic could help avert dual stress for patients of being unable to access clinic personnel and experiencing unwanted AF challenges.

### Limitations

This study has some limitations. First, the findings are limited to patients from only one urban AF clinic, but it has a large reach and overlapping catchment service area with other AF clinics because of its advanced specialized care. Second, despite a robust sample size for a qualitative study, there is potential for selection bias, as recruitment may have attracted participants more positive about their experiences with virtual care and those who had also participated in the larger study survey. Third, the group effect of using focus groups may have limited individual insights, but the relatively small size of the focus groups facilitated more in-depth sharing of individual experiences. Finally, the lack of diversity may limit the transferability of the findings beyond the population represented, although other researchers and users will need to make that determination. However, our sample did represent a similar composition of patients to the clinic demographics.

### Conclusions

Virtual care from a specialty AF clinic provides practical benefits for patients but must be weighed against the need for virtual care’s fit with the complexity of this patient population’s ever-changing needs and problems. Participants clearly defined when virtual care aligned with their needs and when it did not, and this reflected the complexity of their health needs and their management; these are important considerations in decisions about appointment modality. Virtual care fit was often gauged by patients’ perceptions of the effectiveness of their communication with providers and the timeliness of follow-up. Greater attention to the quality and timing of virtual communication may help optimize the fit. Patients recommended that the use of virtual care as a supplement for in-person care in the form of hybrid approaches integrating patient-generated data, video, and digital tools is a better fit with their preferences and needs.

## Appendix

Multimedia Appendix 1 Interview guide.

## Abbreviations

AF: atrial fibrillation
