# Supplementary material for: Patients’ Experiences With the Fit of Virtual Atrial Fibrillation Care During the Pandemic: Qualitative Descriptive Study
Source: JMIR Cardio. 2023 Jan 30;7:e41548. doi: 10.2196/41548 (PMC9926347; doi:10.2196/41548)
Supplement: Multimedia Appendix 1 [file cardio_v7i1e41548_app1.docx]

**
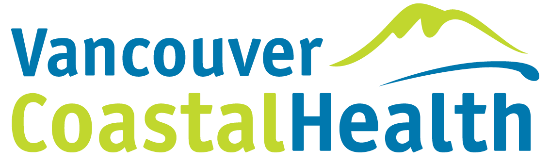
**
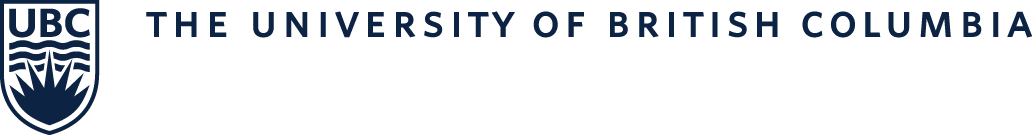


**Multimedia Appendix 1: Interview guide.**

**VIRTUAL ATRIAL FIBRILLATION CLINIC: IMPROVING SPECIALTY CARE DELIVERY**

**Experiences with Virtual Care Interview Script**

**Focus Group Facilitator Instructions**:

Good morning/afternoon/evening. Thank you for taking the time to join us today. My name is __________ and I am a ___<role>____________ on this project. As indicated in the consent form we want to understand your perceptions of using a personal health record. We are conducting several focus groups in the Kootenay Boundary region with patients.

Before we get into our discussion, let me make a few requests:

- First, as outlined in the consent form you should know that we are audio recording the session so that we can refer back to the discussion when we write our report. If anyone is uncomfortable with participating or being recorded please say so and, of course, you are free to leave. We want to assure you of complete confidentiality regarding anything mentioned during our discussion.
- Do speak up and try to have just one person speak at a time to assure that everyone gets a turn.
- Finally, we’re here to exchange opinions and gather the various perspectives on this topic so please say exactly what you think. Don’t worry about what I think or what other members of the group think. The goal is not necessarily to agree or reach consensus, we very much value differing perspectives and would like to hear these.
- If relevant - (Since the group is familiar with one another I’m not going to take the time to introduce ourselves.)
- Our session ends at [insert time] so let’s begin.

**Experience of Virtual Care:**

Can you describe what your experiences have been like using virtual care over the last 6 months?

Looking back on your experiences with usual care (i.e. regular in person visits), can you reflect upon the major differences/similarities you have experienced.

Is there anything you would change about the care you received over the last 6 months?

Have you experienced different forms of virtual care (i.e. phone or zoom)? If so, what form of care would be your preference and why?

**Perceived Quality of Care:**

Can you tell me about any benefits or disadvantages you see to the use of virtual AF care (In what ways has it been better, worse or similar)?

Compared to your in-person care experiences, how well do you feel virtual care is able to allow your care provider to assess your health and concerns

Have there been gaps in your virtual care?

How helpful were the resources (e.g., education, prescriptions, etc.) provided to you during your clinic appointments/care? Are there any other resources you wish you had access to?

**Technology questions:**

Have you had any supports in using the virtual care? If so, what or who has supported you and how? (for example, family helping get you connected, buying equipment for appointments)
